# Syndecan 4 is a marker of endothelial inflammation in pathological aging and predicts long-term cardiovascular outcomes in type 2 diabetes

**DOI:** 10.1186/s13098-024-01431-8

**Published:** 2024-08-20

**Authors:** Angelica Giuliani, Deborah Ramini, Matilde Sbriscia, Paolina Crocco, Luca Tiano, Maria Rita Rippo, Anna Rita Bonfigli, Giuseppina Rose, Maria De Luca, Fabiola Olivieri, Jacopo Sabbatinelli

**Affiliations:** 1 Istituti Clinici Scientifici Maugeri IRCCS, Cardiac Rehabilitation Unit of Bari Institute, Bari, Italy; 2 Clinic of Laboratory and Precision Medicine, IRCCS INRCA, Ancona, Italy; 3 Department of Biology, Ecology and Earth Sciences, University of Calabria, Rende, Italy; 4 Department of Life and Environmental Sciences, Università Politecnica delle Marche, Ancona, Italy; 5 Department of Clinical and Molecular Sciences, Università Politecnica delle Marche, Via Tronto 10/A, 60126 Ancona, Italy; 6 Scientific Direction, IRCCS INRCA, Ancona, Italy; 7 Department of Nutrition Sciences, University of Alabama at Birmingham, Birmingham, AL USA

**Keywords:** Type 2 diabetes, Syndecan 4, Glycocalyx, Major cardiovascular adverse events, Endothelial dysfunction

## Abstract

**Background:**

Endothelial cellular senescence is emerging as a key mechanism of age-related vascular dysfunction. Disruption of the endothelium glycocalyx and shedding of the syndecan (SDC) ectodomains have been associated with several age-related diseases. Although SDC4 is highly expressed in endothelial cells, its levels and shedding in senescent endothelial cells and vascular endothelial dysfunction associated with aging are still unknown.

**Methods:**

To assess whether SDC4 expression was affected by inflammatory conditions, we evaluated its levels in young human umbilical vein endothelial cells (HUVECs) treated with TNF-α at a concentration of 50 ng/mL for 24 h and in cells undergoing replicative senescence. Plasma levels of SDC4 were evaluated in two previously recruited cohorts of (i) subjects with type 2 diabetes (T2D, n = 110) followed for a median of 16.8 years and age- and gender-matched healthy subjects (n = 100), and (ii) middle-aged subjects with mild-to-moderate dyslipidemia. Binomial logistic regression was used to assess whether SDC4 levels could be prognostic for major adverse cardiovascular events (MACE).

**Results:**

In the in vitro study, we showed that HUVECs, when exposed to TNF-α or undergoing replicative senescence, exhibited elevated expression levels of SDC4 and matrix metallopeptidase 9 (MMP-9), as well as increased shedding of SDC4 into the extracellular microenvironment, in comparison to actively proliferating young HUVECs.

Analysis of human samples revealed that patients with T2D without complications exhibited higher SDC4 levels compared to healthy controls and those with T2D vascular complications. In particular, patients with a history of major adverse cardiovascular events (MACE) had lower SDC4 levels. The longitudinal evaluation revealed that higher SDC4 levels predict the onset of new MACE during a 16.8-year follow-up. In the second cohort, no significant association was observed between SDC4 and endothelial dysfunction, assessed by flow-mediated dilation (FMD) or nitric oxide metabolites. SDC4 levels correlated positively with C-reactive protein (CRP) in both cohorts and with PAI-1 in the cohort of patients with T2D.

**Conclusion:**

Overall, we conclude that the shedding of SDC4 from endothelial cells increases under acute (TNF-α treatment) and chronic (senescence) inflammatory conditions and that increased circulating SDC4 levels are associated with systemic inflammation in pathological aging.

**Supplementary Information:**

The online version contains supplementary material available at 10.1186/s13098-024-01431-8.

## Introduction

Endothelial dysfunction is associated with several risk factors for cardiovascular disease (CVD), including arterial hypertension, hypercholesterolemia, and atherosclerosis [15, 43]. Given the diversity in function and heterogeneity of the endothelium, the assessment of endothelial dysfunction presents a challenge, requiring the integration of both laboratory biomarkers and imaging tests to comprehensively evaluate its intricate mechanisms and manifestations [6]. Flow-mediated dilation (FMD) is the most widely used non-invasive technique for examining endothelial function. FMD measures the ability of arteries to induce nitric oxide (NO) release in response to reactive hyperemia [36]. However, its reliability hinges on the operator and may be subject to influence from physiological fluctuations [18]. Direct measurement of endothelial dysfunction is difficult to perform in vivo and, therefore, surrogates must be used [1]. Circulating biomarkers of endothelial activation have surfaced as significant alternatives for conventional methods in diagnosing and stratifying CVD risk and new targets for treatment [30, 33, 34, 44].

A growing body of literature indicates that the glycocalyx covering the luminal endothelial cell surface is a key determinant of the permeability and elasticity of the vascular endothelium [13, 45]. The glycocalyx is composed of highly charged glycolipids, membrane-bound proteoglycans, and sialic acid-containing glycoproteins [26]. Its deterioration, through the shedding of its components in the blood [37], has been proposed to contribute to microvascular dysfunction with advanced age and in the absence of age-related diseases (ARDs) [21].

In endothelial cells, syndecan (SDC)− 3 and − 4 are prominent components of the glycocalyx, with SDC4 expression notably increasing in response to inflammatory stimuli in vitro [42]. SDCs undergo constitutive proteolytic cleavage of their extracellular domain (or ectodomain), a process that is mediated by matrix metalloproteinases (MMPs) [22]. Ectodomain shedding is accelerated in particular conditions, including inflammatory conditions [47] and in response to cardiac injury [38].

Previous work by De Luca and colleagues showed that circulating levels of shed SDC4 were not associated with either TNF-α, IL-6, or arterial elasticity in healthy European-American women older than 60 years [8]. This result entails that inflammation-induced SDC4 shedding might not occur with healthy aging; therefore, in this study, we sought to test the hypothesis that SDC4 may play a role in the vascular alterations occurring with pathological aging, which is associated with a higher pace of senescent cells accrual [46]. To address our hypothesis, we first performed in vitro experiments with umbilical vein endothelial cells (HUVECs) to determine whether the release of SDC4 is induced by TNF-α, as previously seen in human glomerular endothelial cells [31] and whether SDC4 and MMP-9 production and release are altered in senescent HUVECs compared to non-senescent cells.

We then explored the circulating levels of SDC4 in a cohort of patients with type 2 diabetes (T2D), a prototypical ARD that is linked with the accrual of senescent endothelial cells [29] and exhibits a circulating signature that mirrors many of the hallmarks of aging and senescence [35, 39, 41]. We compared SDC4 levels with age- and gender-matched healthy subjects and tested its prognostic value for major adverse cardiovascular events (MACE) over a 16-year follow-up period. Finally, we measured plasma SDC4 levels in a cohort of middle-aged subjects with mild endothelial dysfunction [36].

## Materials and methods

### Cell lines and culture

For the in vitro replicative cell senescence experiments, cryopreserved HUVECs obtained from a pool of donors were purchased from Clonetics (Lonza, Switzerland). HUVECs were cultured in endothelial basal medium (EBM-2, CC-3156, Lonza) supplemented with SingleQuot Bullet Kit (CC-4176, Lonza) to form endothelial growth medium (EGM-2), containing 0.1% human recombinant epidermal growth factor (rh-EGF), 0.04% hydrocortisone, 0.1% vascular endothelial growth factor (VEGF), 0.4% human recombinant fibroblast growth factor (rh-FGF-B), 0.1% insulin-like growth factor-1 with the substitution of arginine for glutamic acid at position 3 (R3- IGF-1), 0.1% ascorbic acid, 0.1% heparin, 0.1% gentamicin and amphotericin-B (GA-1000), and 2% foetal bovine serum (FBS). Cells were seeded at a density of 5000/cm^2^, subcultured when they reached 70–80% confluence, and maintained in a humidified atmosphere of 5% CO^2^ at 37 °C. All cells tested negative for mycoplasma infection. Before replating, harvested cells were counted using a haemocytometer. Population doublings (PDs) were calculated by the formula: (log_10_F–log_10_I) / log_10_2, where F is the number of cells at the end of the passage, and I is the number of seeded cells. Cumulative population doubling (cPD) was calculated as the sum of PD changes. Cells were cultured until the arrest of replication and classified based on SA β-galactosidase (β-gal) activity and telomere length into CON (SA β-Gal < 10%) and SEN (SA β-Gal > 80%) cells. SA β-gal activity was assessed using the Senescence Detection Kit (cat. no. K320, BioVision Inc., USA) as previously described [24].

To investigate the effect of TNF-α on SDC4 and MMP9 in young HUVECs, 50 ng/mL TNF-α was added to EGM-2 for 24 h after adhesion of HUVECs to a 6-well plate. At the end of treatment, the media were collected for the concentration analysis of the released SDC4 through high-sensitivity enzyme-linked immunosorbent assay (ELISA) kits (Human Syndecan-4 Assay kit, cat. no. 27188—Immuno-Biological Laboratories, Minneapolis, MN, USA).

### mRNA expression analysis

Total RNA was isolated from HUVECs employing the Norgen Biotek Kit (Thorold, ON, Canada), according to the manufacturer’s recommendations. RNA was stored at − 80 °C until use. After quantification, 1 µg of RNA was reverse transcribed with PrimeScript RT reagent kit with gDNA eraser (RR047A, Bio. Inc., Shiga, Japan). qPCR reactions were conducted on Rotor Gene Q 5plex HRM (Qiagen) in a 10 μl total reaction volume using TB Green Premix Ex Taq (Tli RNase H Plus). The mRNA expression of the genes of interest was calculated with reference to β-actin. mRNA expression levels were analysed by the 2^−ΔCt^ method. Primer sequences (5ʹ—3ʹ) were as follows: β-actin Fw: AACTGGAACGGTGGTCAAGGTGAC, Rv: CAAGGGACTTCCTGTAACAATGC; SDC4 Fw: CCACGTTTCTAGAGGCGTCACT, Rv: CTGTCCAACAGATGGACATGCT; MMP9 Fw: TATGACATCCTGCAGTGCCC; MMP9 Rv: TTGTATCCGGCAAACTGGCT; p16(ink/4a) Fw: CATAGATGCCGCGGAAGGT, Rv: CTAAGTTTCCCGAGGTTTCTCAGA.

### Immunofluorescent staining

Young and senescent HUVECs were seeded in EGM-2 media at a density of 1 × 10^4^ cells/well on poly-D-lysine coated slides. Cells were washed with PBS and fixed in 4% paraformaldehyde in PBS for 1 h at 4 °C. Cells were washed again in PBS and blocked with 5% BSA for 1 h at room temperature, followed by incubation with SDC4 antibody (5G9) (sc-12766, Santa Cruz Biotechnology, USA) in 1% BSA overnight at 4 °C and with secondary anti-mouse Alexa Fluor 568 antibody (cat. no. A20184, Invitrogen), at room temperature in 1% BSA for 1 h. The actin filaments were labelled with ActinGreen^™^ 488 ReadyProbes^™^ Reagent (R37110, Invitrogen). Cells were stained with nuclear HOECHST 33342 (cat. no. H-3570; Molecular Probes, Oregon, USA) in PBS for 5 min. Finally, cells were cover slipped with Vectashield mounting media (H-1200, Vector Laboratories, Burlingame, CA) and viewed with fluorescence microscopy (Nikon Eclipse 80i, Nikon, Japan). Omission of the primary antibody resulted in a lack of labeling, confirming the specificity of the antibody. The fluorescence intensity of SDC4 staining was quantified in at least 100 cells for each condition and each replicate using the CellProfiler image analysis software, version 4.2.0 [23].

### Study participants

#### Cohort study of T2D

Samples were retrieved from a previously characterized retrospective cohort of 568 patients diagnosed with T2D and 618 healthy controls [4, 41]. The patients were recruited at the Metabolic Diseases and Diabetology Department of IRCCS INRCA between May 2003 and November 2006. For the current investigation, 110 patients with T2D (median age = 68.0 years, interquartile range 62.0–72.0 years) were included. T2D was diagnosed according to American Diabetes Association (ADA) guidelines, i.e., patients having an HbA1C ≥ 6.5% or fasting blood glucose ≥ 126 mg/dl or 2 h blood glucose levels ≥ 200 mg/dl after OGTT, or a random blood glucose ≥ 200 mg/dl when severe diabetes symptoms are present [2]. All subjects provided written informed consent and the original study protocol was approved by the Institutional Review Board of IRCCS INRCA hospital (Approval No. 34/CdB/03). Participants came from Central Italy and provided information such as vital signs, anthropometric measures, medical history, and behavioural data including diet and physical activity. All subjects consumed a Mediterranean diet. The outcome measure was MACE, defined as the nonfatal occurrence of myocardial infarction, cardiac arrest, cardiogenic shock, life-threatening arrhythmia, or stroke in patients without previous history of MACE. Follow-up information was collected from medical records and telephone interviews from the date of enrolment (May 2003–November 2006) to the last day of follow-up (31st December 2019).

#### Cohort study of mild-to-moderate dyslipidemia

Forty-six post-menopausal women (*n* = 27) and men (*n* = 19) aged 49–65 years were tested to assess the correlation between SDC4 and endothelial dysfunction. They were previously enrolled in the QHHC-FMD-PILOT randomized controlled trial, which was conducted at the Italian National Research Center on Aging (INRCA) IRCCS in Ancona, Italy, from December 2016 to June 2017 [36]. The study evaluated the change in endothelium-dependent vasodilation as assessed through FMD of the brachial artery. FMD of the brachial artery was measured ultrasonographically as previously described [36], in adherence with current methodological and physiological guidelines. Serum nitric oxide was also indirectly determined in terms of its products, nitrite, and nitrate (NOx), by the Griess reaction as modified by Miranda et al. [25].

The main inclusion criteria were a BMI between 18.5 and 29.9 kg/m^2^, plasma LDL-C between 130 and 200 mg/dL, and mild endothelial dysfunction defined as FMD between 2.5% and 6.0%. The readers are referred to the original publication of the trial results for additional information about the study protocol and the complete list of inclusion and exclusion criteria [36].

#### SDC4 ELISA

A high-sensitivity enzyme-linked immunosorbent assay (ELISA) kit was used to assess plasma levels of SDC4 (Human Syndecan-4 Assay kit, cat. no. 27188—Immuno-Biological Laboratories, Minneapolis, MN, USA) in samples collected at the time of enrollment in both cohorts.

### Statistical analysis

For all assays on cell cultures, statistically significant (at *p* < 0.05) differences were determined by the two-tailed Student’s t test.

For studies on human subjects, continuous variables were reported as either mean and standard deviation or median and interquartile range based on their distribution (assessed using the Shapiro–Wilk test). For group comparisons, the Mann–Whitney U test and Kruskal–Wallis followed by the Dunn post-hoc test were used. Categorical variables were compared with the χ^2^ test. Correlations between study variables were investigated by bivariate Spearman correlation statistics. Analysis of covariance (ANCOVA) followed by post-hoc tests for multiple comparisons was used to compare the mean differences in SDC4 levels after adjustment for age, sex, and HbA1c. Logistic regression was used to evaluate the associations with the development of MACE, as most of the events were not precisely dated. Significance was accepted as* p* < 0.05. All data were analyzed using the Jamovi software (version 2.3.1) and the SPSS 26.0 for Windows software (SPSS Inc.; Chicago, IL, USA).

## Results

### TNF-α induces release of SDC4 in young HUVECs

**Fig. 1:**
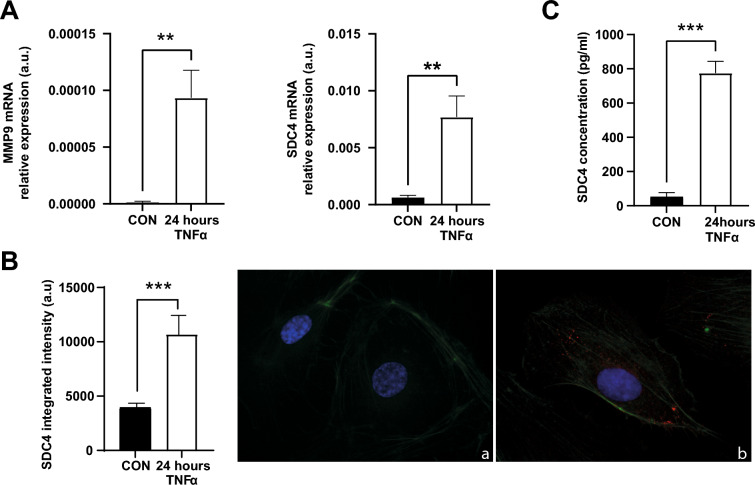
SDC4 levels in TNF-α-treated HUVECs. **A** SDC4 and MMP-9 mRNA relative expression in arbitrary units (a.u.) in HUVECs (CON) and HUVECs treated with TNF-α. **B** Quantification of fluorescence and representative images of immunofluorescence of (a) HUVECs (CON) and HUVECs treated with TNF-α for 24 h, using SDC4 antibody (red fluorescence). Nuclear DNA was labelled with HOECHST (blue), while β-actin was labelled ActinGreen 488 ReadyProbes (green fluorescence). **C** SDC4 concentration (pg/mL) in the culture medium of HUVECs (CON) and HUVECs treated with TNF-α. Data are the mean and SD of three independent experiments. *, p < 0.05 for paired Student’s *t* tests

In the first series of in vitro experiments, we confirmed that administration of TNF-α induces a higher *SDC4* and *MMP-9* mRNA expression (Fig. 1A) and increases SDC4 protein expression (Fig. 1B) in young HUVECs. We also observed a significantly higher release of SDC4 in 24-h conditioned media after administration of TNF-α (Fig. 1C), suggesting an increased SDC4 shedding.

### Endothelial production and release of SDC4 is enhanced by replicative cell senescence

**Fig. 2:**
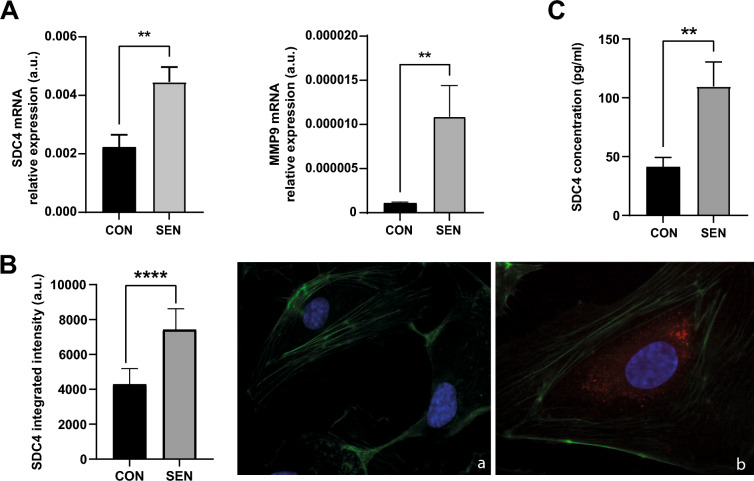
SDC4 levels in senescent HUVECs.** A** SDC4 and MMP-9 relative expression in arbitrary units (a.u.) in young HUVECs (CON) and senescent HUVECs (SEN). **B** Quantification of fluorescence and representative images of immunofluorescence of (a) young HUVECs (CON) and senescent HUVECs (HUVECs), using SDC4 antibody (red fluorescence). Nuclear DNA was labelled with HOECHST (blue), while β-actin was labelled ActinGreen 488 ReadyProbes (green fluorescence). **C** SDC4 concentration (pg/mL) in the culture medium of young HUVECs (CON) and senescent HUVECs (SEN). Data are mean and SD of three independent experiments. *, p < 0.05 for paired Student’s *t* tests

Next, we used an in vitro model of replicative cell senescence to gain insight into the potential effect of aging on SDC4 shedding in endothelial cells. After verifying that Sen HUVECs were growth-arrested, as indicated by reduced cPDs, up-regulation of *p16* mRNA expression, and increased SA β-gal activity (Supplementary Fig. 1), we examined SDC4 production and release. As shown in Fig. 2, compared to Con cells, Sen cells were characterized by a higher *SDC4* mRNA expression (*p* = 0.0042; Fig. 2A) and a higher SDC4 immunofluorescence intensity (*p* < 0.001; Fig. 2B). Moreover, analysis of SDC4 in conditioned media after 24 h of incubation revealed that Sen cells released a significantly greater amount of SDC4 compared to proliferating cells (*p* = 0.0060; Fig. 2C). Consistently with a higher shedding of SDC4, Sen cells also showed a greater expression of MMP-9 (*p* = 0.0084; Fig. 2A).

### Circulating SDC4 is a prognostic biomarker of cardiovascular events

Baseline demographic and biochemical characteristics of 110 patients with type 2 diabetes and 100 age- and gender-matched healthy controls are reported in Table 1. Significant differences were observed for different biochemical variables, i.e. BMI, Waist-hip ratio, HDL-cholesterol, fasting glucose, glycated hemoglobin, eGFR, and C reactive protein.

**Table 1:** Biochemical and anthropometric characteristics of healthy control subjects (CTR) and patients with type 2 diabetes mellitus (T2D)

Variables	Healthy control (n = 100)	Type 2 Diabetes (n = 110)	p-value
Age (years)	66.0 (57.8–73.3)	68.0 (62.0–72.0)	0.823
Gender (males, %)	59 (59%)	61 (55.5%)	0.604
BMI (Kg/m^2^)	26.9 (24.8–29.8)	28 (26.0–31-0)	**0.020**
Weight (Kg)	75.0 (66.0–83.0)	77.0 (69.0–85.0)	0.137
Waist-hip ratio	0.915 (0.860–0.967)	0.940 (0.896–0.969)	**0.030**
Total cholesterol (mg/dL)	209 (183–231)	198 (179–226)	0.242
HDL-C (mg/dL)	55.0 (45.0–62.0)	47.5 (42.3–58.8)	**0.010**
LDL-C (mg/dL)	117 (94.9–138)	114 (91.9–136)	0.482
Triglycerides (mg/dL)	92 (72.0–139)	116 (77.0–155)	0.080
Glucose (mg/dL)	92.5 (87.8–100.0)	146.0 (126–174)	** < 0.001**
HbA1C (%)	5.60 (5.40–5.90)	7.20 (6.60–8.07)	** < 0.001**
Insulin (µUI/mL)	5.59 (3.89–7.62)	5.80 (4.24–8.72)	0.431
HOMA index	1.27 (0.90–1.79)	2.07 (1.41–3.40)	** < 0.001**
Hemoglobin (g/dL)	14.2 (13.6–15.1)	14.2 (13.6–14.9)	0.632
White blood cells (10^3^/µL)	6.02 (4.96–6.85)	6.32 (5.43–7.25)	0.068
Neutrophil-to-lymphocyte ratio (NLR)	1.59 (1.19–2.15)	1.85 (1.47–2.41)	**0.026**
Ferritin (ng/mL)	68.5 (36.7–150.2)	82.1 (36.7–146.3)	0.777
hs-CRP (mg/L)	1.88 (0.97–4.08)	3.08 (1.33–5.67)	**0.018**
Interleukin-6 (pg/mL)	0.25 (0.07–0.48)	0.37 (0.13–0.80)	0.235
PAI-1 (ng/mL)	19.7 (13.8–29.0)	19.1 (15.7–26.5)	0.602
eGFR	81.6 (69.9–95.7)	73.8 (63.6–85.3)	**0.020**
Syndecan-4 (pg/mL)	76.7 (44.9–106.1)	77.4 (46.7–125.2)	0.353
Disease duration (years)	–	13 (6–20)	–
Relevant medications (n, %)			
Metformin	–	40 (36.4%)	–
Sulphonylureas	–	39 (35.5%)	–
Glinides	–	8 (7.3%)	–
Insulin	–	25 (22.7%)	–
Statins	–	16 (14.5%)	–
Vitamin K antagonists	–	29 (26.4%)	–
T2D complications (n)			
Retinopathy	–	25 (22.7%)	–
Nephropathy	–	12 (10.9%)	–
Neuropathy	–	16 (14.5%)	–
History of MACE	–	17 (15.5%)	–
Peripheral artery disease	–	16 (14.5%)	–
Any complication	–	57 (51.8%)	–

Significant p-values are reported in bold

**Fig. 3:**
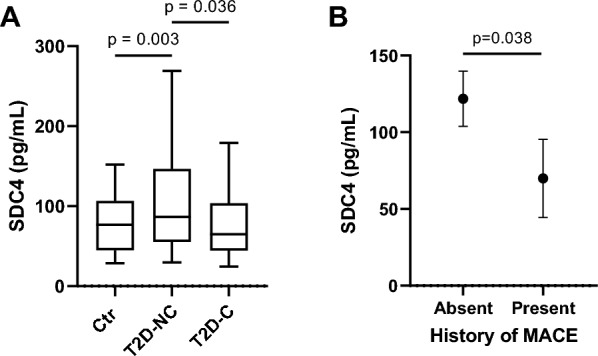
SDC4 levels in type 2 diabetes. **A** Levels of SDC4 in healthy subjects and in T2D with (T2D-C) or without (T2D-NC) complications. P values for post-hoc tests following one-way ANOVA. **B** Marginal mean plot of serum SDC4 in patients with type 2 diabetes grouped according to the presence of MACE. P values for Tukey’s post-hoc tests following one-way ANCOVA

There were no significant differences in SDC4 levels between CTR and T2D (*p* = 0.353); therefore, we evaluated its differential expression in patients with T2D grouped according to the presence (T2D-C, *n* = 57) or absence (T2D-NC, *n* = 53) of complications. Interestingly, we found that SDC4 levels were higher in T2D-NC compared to healthy subjects (*p* = 0.003) and T2D-C (*p* = 0.036) (Fig. 3A). On the other hand, no difference was observed between T2D-C and CTR (*p* = 0.823) (Fig. 3A). No significant sex-related differences were observed for SDC4 levels (p = 0.455, data not shown).

To explore the effect of the presence of micro- and macrovascular complications of diabetes, i.e., neuropathy, nephropathy, retinopathy, peripheral vascular disease, and MACE, a multivariable ANCOVA, was performed using SDC4 levels as the dependent variable, after adjustment for age, HbA1c, and the presence of T2D complications (Table 2). Serum SDC4 was significantly lower in patients with a history of MACE (*p* = 0.038; Fig. 3B), while the other complications did not affect SDC4 levels. We hypothesized that the amelioration of SDC4 levels in patients who experienced a MACE could be related to more strict pharmacological therapy and control of risk factors in secondary prevention. Moreover, it has been reported that antihypertensive drugs affect levels of SDC4, with β-blockers significantly decreasing the levels of circulating SDC4 [20]. Thus, we next compared the proportion of subjects under specific therapies according to the history of MACE. As predicted, subjects that suffered from a previous MACE were more likely to be on anti-hypertensive (20.5% vs. 6.5%, *p* < 0.001), vitamin K antagonists (23.0% vs. 13.6%, *p* = 0.033), and lipid-lowering drugs (25.8% vs. 11.7%, *p* < 0.001). No significant association was observed between serum SDC4 and blood glucose control assessed in terms of HbA1c (Spearman’s rho = − 0.08, p = 0.392) or glucose-lowering treatments at baseline (data not shown).

**Table 2:** Circulating levels of SDC4 in patients with type 2 diabetes mellitus (T2DM) in relation to the different T2DM-related complications

Variable	Sum of squares	gdl	Mean square	F	p-value
Age (years)	37.1	1	37.1	0.00477	0.945
HbA1c	3157.9	1	3157.9	0.40587	0.526
History of MACE	34,496.0	1	34,496.0	4.43357	0.038
Peripheral artery disease	13,904.1	1	13,904.1	1.78701	0.184
Nephropathy	1010.9	1	1010.9	0.12992	0.719
Retinopathy	12,959.6	1	12,959.6	1.66562	0.200
Neuropathy	65.8	1	65.8	0.00845	0.927

*MACE* major adverse cardiovascular events

P-values derived from post-hoc tests with Tukey's corrections after analysis of covariance (ANCOVA). Age, and HbA1c (haemoglobin A1C) are considered as covariates

A binomial logistic regression, adjusted for sex, age, BMI, HbA1c, LDL-C, hs-CRP, Troponin I, NT-proBNP, presence of hypertension, and lipid-lowering therapy confirmed that lower SDC4 is associated with history of MACE (Table 3).

**Table 3:** Logistic regression model for the association with history of MACE in patients with T2D

Predictor	Estimate (SE)	Z	p-value	OR (95% CI)
SDC4 (10 pg/mL increase)	− 0.218 (0.091)	− 2.387	0.017	0.804 (0.672–0.962)
Age (years)	0.118 (0.067)	1.767	0.077	1.126 (0.987–1.283)
Sex (Male)	0.199 (0.783)	0.254	0.799	1.221 (0.263–5.667)
HbA1c (%)	− 0.147 (0.269)	− 0.545	0.586	0.864 (0.509–1.464)
LDL-C (mg/dL)	− 0.0041 (0.012)	− 0.3395	0.734	0.9959 (0.972–1.020)
hs-CRP (mg/L)	− 0.036 (0.062)	− 0.586	0.558	0.965 (0.855–1.089)
Troponin I (ng/L)	− 0.058 (0.054)	− 1.067	0.286	0.944 (0.848–1.05)
NT-proBNP (ng/L)	0.002 (0.001)	1.380	0.167	1.002 (0.999–1.004)
Hypertension	1.846 (1.133)	1.6289	0.103	6.336 (0.687–58.421)
Statin Therapy	0.826 (0.795)	1.039	0.299	2.284 (0.481–10.846)

Model summary, *p* < 0.001, Nagelkerke’s R^2^ = 0.312

Next, we explored the correlation between SDC4 and CRP, a general marker of systemic inflammation in subjects with T2D. We found a statistically significant positive correlation between plasma SDC4 levels and the acute phase proteins CRP (Spearman’s rho = 0.257; *p* = 0.013) and PAI-1 (Spearman’s rho = 0.205; *p* = 0.034), after controlling for age and gender. No significant correlations were evidenced between SDC4 and other markers of systemic inflammation available in our cohort, including ferritin, interleukin-6, white blood cells, and neutrophil-to-lymphocyte ratio (NLR) (data not shown).

Finally, we evaluated the prognostic value of SDC4 for the development of MACE during a 16.8-year follow-up. A binomial logistic regression analysis, adjusted for age, sex, HbA1c, LDL-C, lipid-lowering therapy, hs-CRP, the presence of hypertension, Troponin I and NT-proBNP revealed that higher SDC4 is independently associated with increased odds of developing a MACE (for 10 pg/ml of SDC4 increase, OR: 1.08, 95% CI 1.02–1.56; Table 4). As expected, male sex and increasing HbA1c were observed as significant predictors of MACE.

**Table 4:** Logistic regression model predicting likelihood of developing MACE in patients with T2D without previous history of MACE

Predictor	Estimate (SE)	Z	p-value	OR (95% CI)
SDC4 (10 pg/mL increase)	0.079 (0.033)	2.397	0.017	1.082 (1.015–1.155)
Age (years)	0.012 (0.039)	0.312	0.755	1.012 (0.937–1.094)
Sex (Male)	1.434 (0.718)	1.998	0.046	4.195 (1.028–17.119)
HbA1c (%)	0.568 (0.251)	2.265	0.024	1.765 (1.08–2.887)
LDL-C (mg/dL)	− 0.013 (0.011)	− 1.152	0.249	0.987 (0.965–1.01)
hs-CRP (mg/L)	0.048 (0.043)	1.121	0.262	1.049 (0.965–1.141)
Troponin I (ng/L)	− 0.105 (0.055)	− 1.889	0.059	0.901 (0.808–1.004)
NT-proBNP (ng/L)	0.002 (0.002)	1.212	0.225	1.002 (0.999–1.006)
Hypertension	0.893 (0.631)	1.415	0.157	2.442 (0.709–8.416)
Statin Therapy	0.6504 (0.779)	0.8353	0.404	1.916 (0.417–8.816)

Model summary, p < 0.001, Nagelkerke’s R^2^ = 0.225

### SDC4 levels correlate with low-grade systemic inflammation but not with endothelial dysfunction

**Fig. 4:**
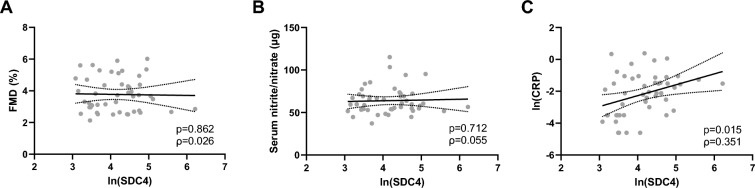
Circulating SDC4 levels do not correlate with endothelial dysfunction in healthy middle-aged Italian subjects. **A**–**B** Scatterplots showing the correlation of plasma levels of SDC4 with flow-mediated dilation (FMD) (panel **A**), nitric oxide metabolites (panel **B**) and C-reactive protein (CRP) (panel **C**)

Next, we investigated whether SDC4 levels might be associated with endothelial-dependent vasodilation in a cohort of middle-aged Italian subjects. The demographic and biochemical variables of the subjects used in this part of the study are reported in Supplementary Table 1. There was no significant correlation between plasma levels of SDC4 and FMD (Fig. 4A) or NO metabolites (Fig. 4B) in this cohort. On the other hand, a positive correlation (Spearman’s rho = 0.351;* p* = 0.014) between SDC4 and CRP was also found in this cohort (Fig. 4C).

## Discussion

SDC4 is a key component of the endothelial glycocalyx [32] and exerts relevant functions in endothelial activation or dysfunction promoted by a variety of conditions, such as inflammation and mechanical stress from pulsatile blood flow, both in treated endothelial cells and in vivo [3, 9, 10, 27, 31, 42]. Vuong and collaborators were the first to report that, together with SDC3, SDC4 is highly expressed in primary HUVECs, and its expression is rapidly and strongly increased by treatment of the cells with the inflammatory mediators, lipopolysaccharide (LPS) and interleukin-1β (IL-1β) [42]. Consistently, in the present study, we showed that intracellular and extracellular SDC4 levels are increased in endothelial cells under acute and chronic pro-inflammatory conditions, which were recapitulated by exposure to TNF-α and replicative senescence, respectively.

The increased burden of senescent cells that occurs in most tissues during aging is a major driver of the chronic proinflammatory state that contributes to age-related diseases, including type 2 diabetes and its micro- and macro-vascular complications [28]. Senescent endothelial cells contribute to the development of endothelial dysfunction [14, 19] also through the mediation of glycocalyx impairment [16]. However, to our knowledge, no studies have evaluated SDC4 expression in senescent endothelial cells. SDC4 is essential for sensing flow direction and for inhibiting of atherosclerotic lesion formation [3]. More recently, Chala and collaborators [5] reported that Sen HUVECs exhibit heightened mechanical interaction with the in vitro substrate due to a significant rise in basal adhesion and force generation facilitated by robust focal adhesions.

However, this heightened mechanical interaction compromises their capacity to adapt to local hemodynamic conditions [5]. Considering the involvement of SDC4 in the assembly of focal adhesions in HUVECs [42], it is plausible that its enhanced production in Sen HUVECs observed in our study might be explained by its participation in this process. Furthermore, it is quite convincing that the proinflammatory microenvironment surrounding senescent cells can play a role in the synthesis and shedding of SDC4.

Additionally, we showed that the expression of MMP-9 is significantly higher after exposure of HUVECs to TNF-α, as previously observed in conditionally immortalized human glomerular endothelial cells [31]. Interestingly, the blockade of TNF-α by the immunomodulatory agent infliximab ameliorated SDC4 shedding in human cardiomyocytes [40]. Altogether, these findings from independent in vitro studies strongly indicate that the production and MMP-9-mediated shedding of SDC4 are crucial in the response of endothelial cells to inflammation. This agrees with the well-recognized role of heparan sulfate proteoglycans (HSPGs) in the events that occur during inflammation [12].

Motivated by our in vitro findings we sought to investigate whether serum levels of SDC4 are affected by the presence of T2D, one of the most prevalent age-related chronic inflammatory diseases. One interesting finding of this component of the study is that subjects with T2D without complications had higher levels of SDC4 compared not only with healthy controls but also with patients having at least one T2D complication. Going deeper into the complications of diabetes, we found that lower SDC4 levels were associated with the history of MACE while higher levels predicted new onset of MACE, during a long-term follow-up (16.8 years), independently of conventional CV risk factors and biomarkers of myocardial injury, i.e. troponin I and NT-proBNP. Notably, high levels of SDC4 were previously associated with CV mortality in hemodialysis patients, including subjects with T2D [17]. Our findings might reflect the impact of aggressive pharmacological management on subjects with T2D who experienced a complication, which is particularly relevant in the secondary prevention of MACE, as observed in our cohort, compared to individuals without complications. It is conceivable that proper management of CV risk factors may reduce SDC4 shedding by decreasing the burden of systemic inflammation, a hypothesis that deserves further investigation.

In this study, we did not observe a significant association of plasma levels of SDC4 with either endothelial dysfunction, assessed in terms of FMD, nor NO metabolites in middle-aged healthy subjects. Previously, we reported evidence of a role for SDC4 in the physiological regulation of BP in both healthy premenopausal and postmenopausal women [7]. The lack of correlation herein reported was probably due to the limited sample size and variability of the study population, which consisted of subjects with mild-to-moderate dyslipidemia and no comorbidities. However, SDC4 levels were positively related with CRP in both cohorts of healthy and T2D subjects SDC4, suggesting its involvement in systemic inflammation [11, 47].

The present study has limitations that need to be addressed. First, although we were able to demonstrate both increased synthesis and release of SDC4 by cells exposed to TNF-α, we did not provide mechanistic evidence connecting the heightened shedding of SDC4 to endothelial activation. Second, progress in the pharmacological management of T2D during the long follow-up period that may have affected the CVD outcomes should be regarded as potential confounders when evaluating the predictive role of SDC4. Third, we were unable to draw definitive conclusions on the correlation between SDC4 and endothelial dysfunction assessed in terms of FMD. However, we believe that the availability of long-term cardiovascular outcome data may provide a useful tool to identify biomarkers that could explain the additional components of CV risk that are not captured by conventional risk factors.

In conclusion, dysregulation of SDC4 expression or function may contribute to the pathogenesis of conditions such as atherosclerosis, hypertension, and myocardial infarction. Further studies will verify circulating SDC4 age-related trends both in healthy people of different ages and in patients affected by the most common age-related diseases (ARDs) and/or major risk factors associated with the development of ARDs.

### Supplementary Information

Supplementary material 1.

## Data Availability

The data that support the findings of this study are available on request from the corresponding author.
