# Supplementary material for: Syndecan 4 is a marker of endothelial inflammation in pathological aging and predicts long-term cardiovascular outcomes in type 2 diabetes
Source: Diabetol Metab Syndr. 2024 Aug 20;16:203. doi: 10.1186/s13098-024-01431-8 (PMC11334569; doi:10.1186/s13098-024-01431-8)
Supplement: Supplementary file 1 — Supplementary material 1. [file 13098_2024_1431_MOESM1_ESM.pdf]

Supplementary Figure 1. Characterization of senescent HUVECs. (A) Cumulative population doublings (cPDs) curve of HUVECs undergoing replicative senescence (X axis: cell passages from P1 to P17). **(B)** mRNA relative expression of p16 in young (CON) and in senescent HUVECs (SEN). **(C)** Representative positivity and quantification of senescence-associated β-galactosidase (SA β-gal) in (a) young HUVECs (CON, <10%) and (b) senescent (SEN, >80%) cells.

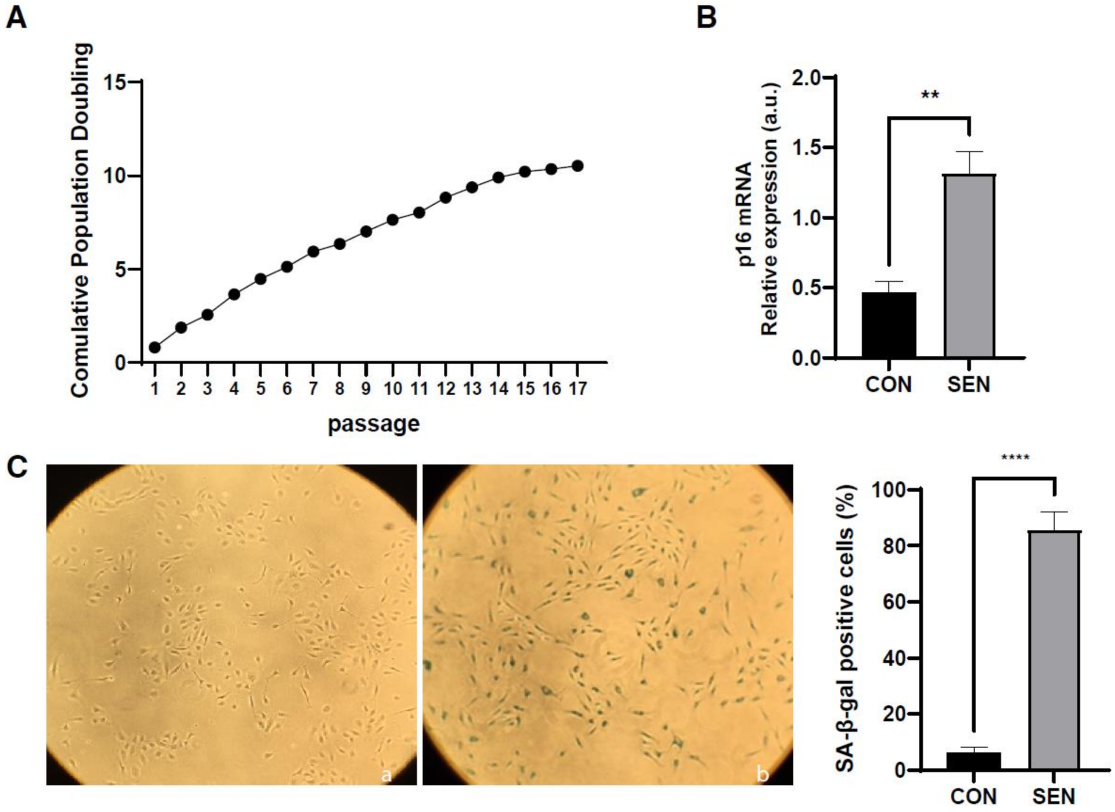

Supplementary Table 1. Baseline clinical and demographic characteristics of the 48 subjects of the QHHC-FMD-PILOT cohort.

**Variables**	**Mean (SD)**
Age (years)	58.9 (5.1)
Gender (males, %)	21 (44%)
FMD (%)	3.79 (1.09)
SDC4 (pg/mL)	82.2 (78.4)
BMI (Kg/m^2)	24.2 (3.3)
Total cholesterol (mg/dL)	220.8 (32.0)
LDL cholesterol (mg/dL)	151.7 (20.6)
HDL cholesterol (mg/dL)	61.1 (15.9)
Triglycerides (mg/dL)	114.4 (69.1)
Glucose (mg/dL)	95.7 (9.8)
HOMA index	1.27 (0.57)
Hemoglobin (mg/dL)	14.4 (1.0)
WBC (cells/mm3)	6.57 (1.17)
Creatinine (mg/dL)	0.85 (0.16)
Alanine aminotransferase (U/L)	19.3 (8.7)
hs-CRP (mg/L)	0.2 (0.3)
ESR (mm/h)	14.1 (8.9)

FMD, flow mediated dilation; BMI, SDC4, syndecan 4; body mass index; WBC, white blood cells; LDL, low-density lipoprotein; HDL, high-density lipoprotein; HOMA, homeostatic model assessment; hs-CRP, high sensitivity C-reactive protein; ESR, erythrocyte sedimentation rate.
